# Gallbladder Perforation Treated Using a Combination of Percutaneous Transhepatic and Endoscopic Transpapillary Gallbladder Drainage

**DOI:** 10.7759/cureus.108618

**Published:** 2026-05-10

**Authors:** Kanta Niinomi, Shinji Monoe, Ryo Nishio, Yu Yasue, Ryota Hirao

**Affiliations:** 1 Department of Gastroenterology, Nakatsugawa Municipal General Hospital, Nakatsugawa, JPN; 2 General Practice, Toyota Regional Medical Center, Toyota, JPN

**Keywords:** acute cholecystitis, combined drainage, endoscopic transpapillary gallbladder drainage, ercp, gallbladder perforation, hepatic abscess, percutaneous transhepatic gallbladder drainage, subcapsular hepatic abscess

## Abstract

Gallbladder perforation is an uncommon but serious complication of acute cholecystitis. When associated with a subcapsular hepatic abscess, definitive surgical management is typically recommended; however, surgery and direct abscess drainage may not be feasible in patients with high operative risk or unfavorable anatomy. Percutaneous transhepatic gallbladder drainage (PTGBD) provides external decompression of the gallbladder, whereas endoscopic transpapillary gallbladder drainage (ETGBD) provides internal drainage through the cystic duct. We report the case of an 88-year-old man with acute cholecystitis complicated by gallbladder perforation and a subcapsular hepatic abscess. Computed tomography revealed gallbladder distension and a subcapsular low-attenuation area along the anterior liver surface. Magnetic resonance cholangiopancreatography demonstrated communication between the gallbladder and the subcapsular collection. Emergent cholecystectomy was contraindicated because of advanced age, reduced activities of daily living, renal dysfunction, cerebrovascular disease, and respiratory dysfunction. Direct percutaneous abscess drainage was also not feasible because of intervening pulmonary and vascular structures. Although PTGBD initially improved the inflammatory response, C-reactive protein levels subsequently rebounded, indicating inadequate infection control. Cholangiography via the PTGBD catheter confirmed contrast leakage from the gallbladder fundus into the subcapsular cavity. ETGBD was therefore performed to enhance internal drainage through this communication, and a 5-Fr plastic stent was successfully placed with the aid of a novel sphincterotome. Following the procedure, inflammatory markers normalized, and follow-up imaging confirmed abscess resolution. The patient was discharged on day 56 without recurrence. This case suggests that combined PTGBD and ETGBD may be an effective non-operative strategy in selected high-risk patients with gallbladder perforation and a communicating subcapsular hepatic abscess when surgery and direct abscess drainage are not feasible.

## Introduction

Acute cholecystitis is a common biliary infection, but severe inflammation may occasionally lead to gallbladder wall ischemia, necrosis, and perforation [1,2]. In rare cases, perforation may be associated with abscess formation extending into adjacent tissues, including the liver [2,3].

Subcapsular hepatic abscess associated with gallbladder perforation is an uncommon but clinically important complication. Because retained gallstones, persistent infection, and recurrent inflammation remain concerns, surgical management is often preferred when feasible [4]. However, some patients are not suitable surgical candidates because of advanced age, multiple comorbidities, or poor general condition. In addition, direct percutaneous drainage of the abscess may be technically challenging when a safe access route cannot be established.

Percutaneous transhepatic gallbladder drainage (PTGBD) is an established method for gallbladder decompression, involving an external drainage catheter placed percutaneously through the liver [4]. Endoscopic transpapillary gallbladder drainage (ETGBD), which includes endoscopic naso-gallbladder drainage and endoscopic gallbladder stenting (EGBS), achieves internal drainage through the cystic duct during endoscopic retrograde cholangiopancreatography (ERCP) and has emerged as an alternative or adjunctive option in selected high-risk surgical patients [5]. In a prospective randomized study, EGBS achieved a technical success rate of 86.1% and a per-protocol clinical success rate of 90.3%; however, selective cystic duct cannulation remains technically challenging because of anatomical factors [5,6]. In cases where a communication exists between the gallbladder lumen and an adjacent abscess cavity, a combined PTGBD and ETGBD approach may provide complementary external and internal drainage. This dual-modality strategy can enhance gallbladder decompression and facilitate effective drainage of the communicating abscess cavity, particularly when both surgery and direct percutaneous abscess drainage are not feasible.

In a PubMed search performed on February 10, 2026, using the terms “gallbladder perforation,” “hepatic abscess,” and “endoscopic transpapillary gallbladder drainage,” we did not identify any prior reports describing a combined PTGBD-ETGBD strategy to treat this complication.

Herein, we report the case of an 88-year-old male patient in whom effective disease control was achieved using this combined approach, facilitated by a novel sphincterotome.

## Case presentation

An 88-year-old male patient with a history of cerebral infarction presented with acute right upper quadrant pain. On admission, he was febrile (38.6 °C), with a blood pressure of 102/66 mmHg, heart rate of 120 beats/min, and respiratory rate of 18 breaths/min. Physical examination revealed right costal tenderness with a positive Murphy’s sign, and his level of consciousness corresponded to a Japan Coma Scale score of 3. Laboratory evaluation demonstrated severe inflammatory and hepatobiliary abnormalities (Table 1).

**Table 1:** Laboratory findings on admission. WBC, white blood cell count; CRP, C-reactive protein; AST, aspartate aminotransferase; ALT, alanine aminotransferase; ALP, alkaline phosphatase; γ-GTP, gamma-glutamyl transpeptidase; BUN, blood urea nitrogen.

Parameter	Result	Reference range
WBC (/µL)	14,800	4,500-8,500
Neutrophils (%)	88.4	40-75
Platelets (×10⁴/µL)	17.1	14-35
CRP (mg/dL)	29.95	<0.3
Total bilirubin (mg/dL)	4.4	0.2-1.3
AST (U/L)	85	8-38
ALT (U/L)	88	4-44
ALP (U/L)	210	38-113
γ-GTP (U/L)	78	0-70
BUN (mg/dL)	62.7	8.0-20.0
Creatinine (mg/dL)	2.08	0.6-1.1
Albumin (g/dL)	3	3.8-5.2

**Figure 1:**
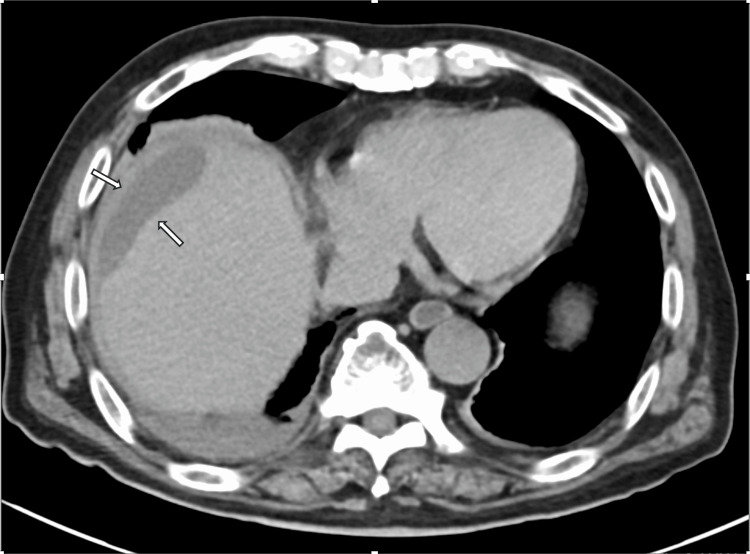
Non-contrast abdominal computed tomography showing gallbladder distension and pericholecystic fat stranding. Arrows indicate a subcapsular low-attenuation area in the anterior liver.

Abdominal computed tomography (CT) showed gallbladder distension with prominent pericholecystic fat stranding, alongside a low-attenuation area measuring ~81 × 12 mm beneath the anterior liver (Figure 1).

**Figure 2:**
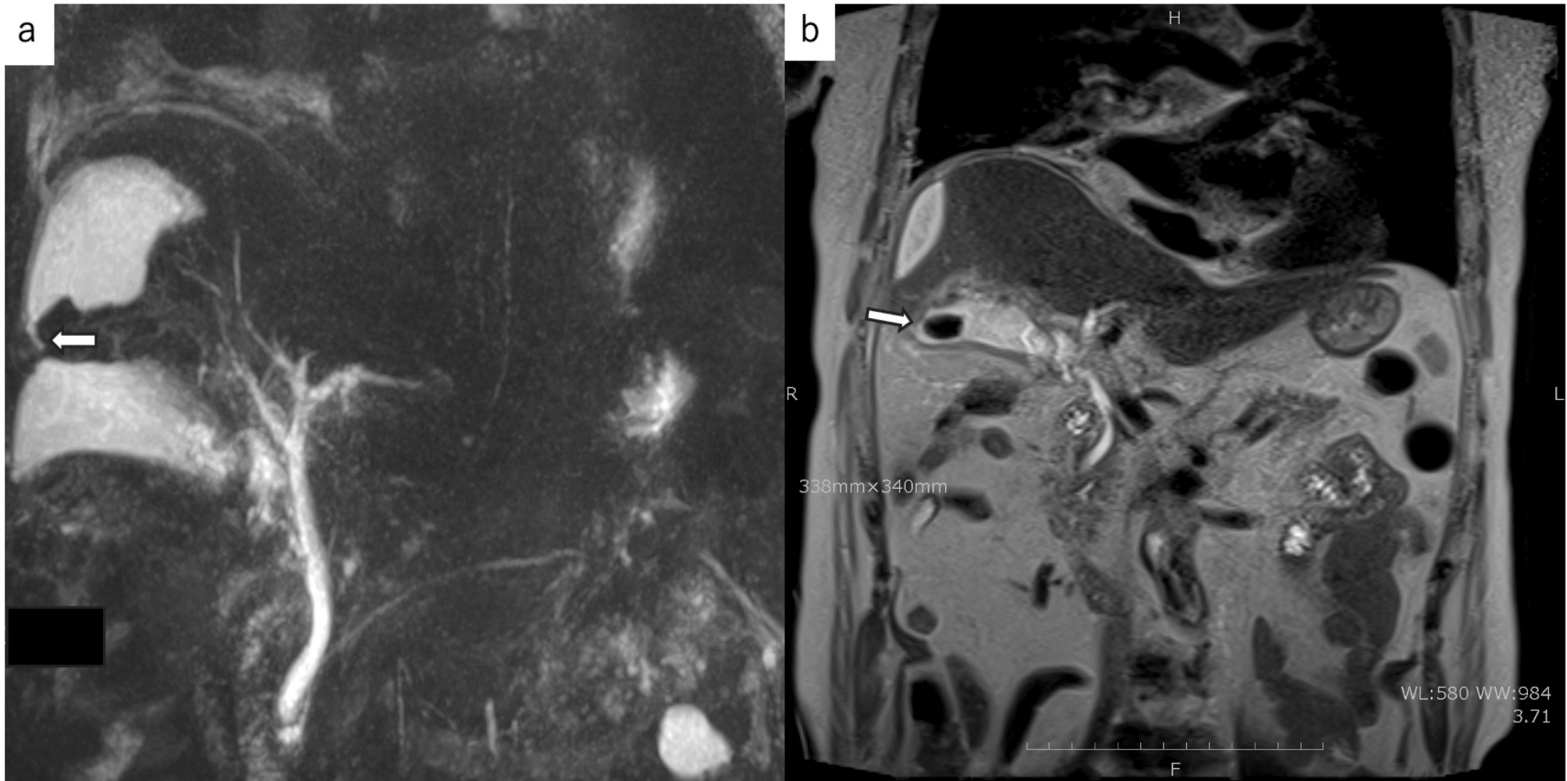
Magnetic resonance cholangiopancreatography. (a) Arrow indicates communication between the gallbladder and the subcapsular abscess cavity in the anterior liver. (b) Arrow indicates a gallstone within the gallbladder.

Magnetic resonance cholangiopancreatography further demonstrated a direct communication between the gallbladder lumen and subcapsular hepatic abscess cavity, as well as multiple gallstones (Figure 2).

Based on these findings, the patient was diagnosed with gallbladder perforation complicated by a subcapsular hepatic abscess secondary to acute cholecystitis, classified as Grade III according to the Tokyo Guidelines 2018 [7]. These findings were considered atypical for routine acute cholecystitis because the diagnosis depended primarily on imaging evidence showing subcapsular extension and fistulous communication, rather than symptoms alone.

The patient was of advanced age, required a cane for ambulation, had reduced activities of daily living, a serum creatinine level >2 mg/dL on admission, and underlying cerebrovascular disease. Given the prohibitively high operative risk, emergent surgery was considered contraindicated. The patient was therefore hospitalized, and PTGBD was scheduled for the same day. Ultrasonography revealed biliary sludge within the gallbladder and confirmed the absence of intervening vascular structures along the planned puncture route. The gallbladder was punctured using an 18 G needle, yielding turbid bile on aspiration. A 7 Fr pigtail catheter was subsequently placed, and external drainage was initiated. Percutaneous transhepatic abscess drainage (PTAD) of the subcapsular hepatic abscesses was also considered; however, no safe access route was identified because the abscess cavity was partially covered by the lung and surrounding vascular structures. Antibiotic therapy was initiated, which produced an initial improvement in the patient’s inflammatory markers, hepatobiliary enzymes, and renal function. Blood cultures were negative, whereas bile cultures grew Klebsiella pneumoniae and Klebsiella oxytoca.

Subsequent cholangiography demonstrated contrast leakage from the gallbladder’s fundus into the subcapsular abscess cavity, confirming fistulous communication. As the patient’s respiratory function deteriorated further, both the surgical and pulmonology teams concluded that operative intervention was infeasible. Despite PTGBD placement, drainage output remained limited, with persistently purulent bile. Saline irrigation flowed smoothly without catheter occlusion. Drainage volume gradually decreased, and irrigation yielded only minimal amounts of purulent bile. The patient’s C-reactive protein (CRP) level decreased to 0.40 mg/dL on day 22 of his hospitalization, but subsequently rebounded to 5.89 mg/dL by day 35, indicating inadequate inflammation control.

**Figure 3:**
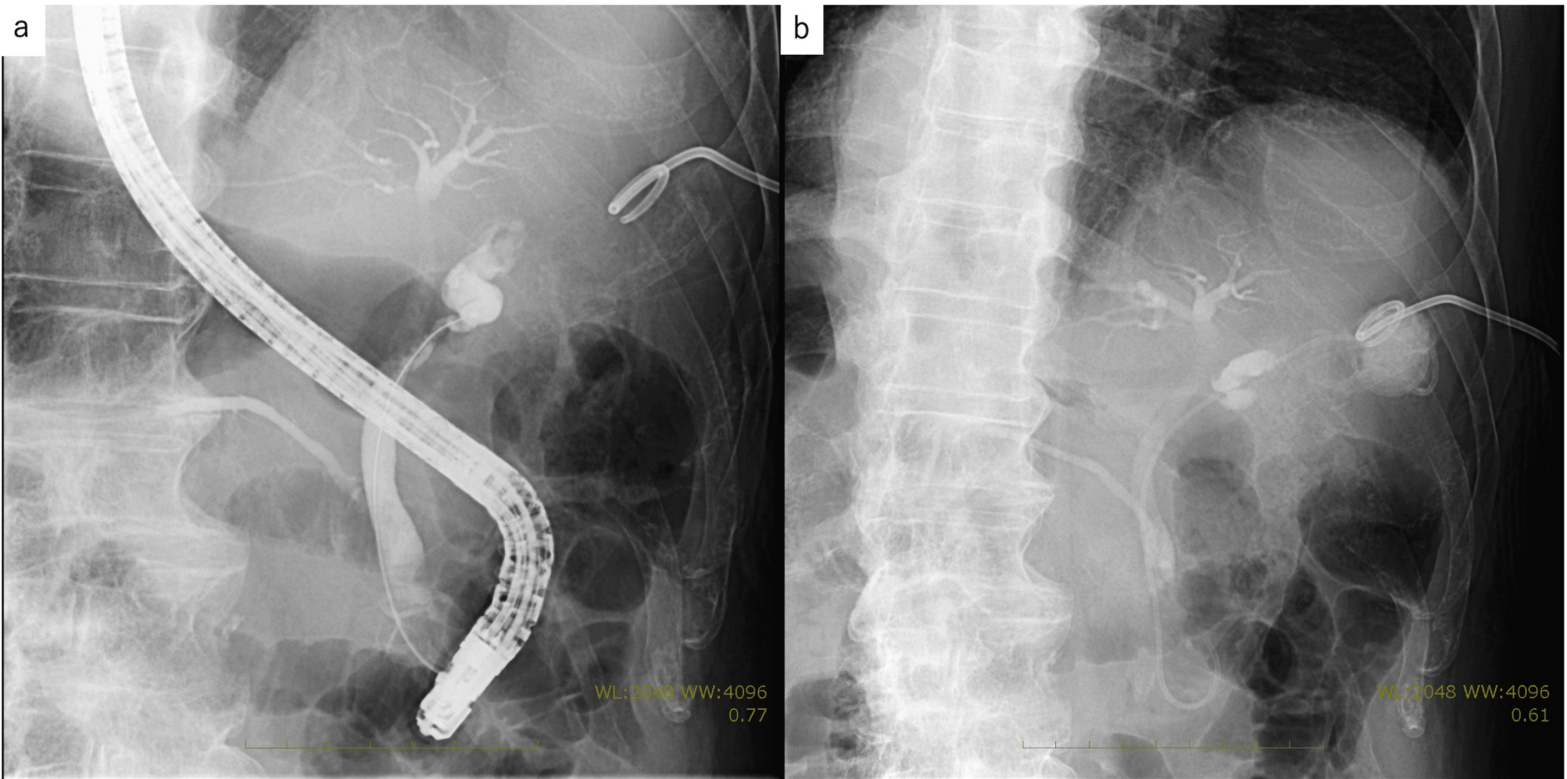
Endoscopic retrograde cholangiopancreatography (ERCP) images. (a) Selective guidewire cannulation of the cystic duct using Engetsu (Kaneka Medix, Osaka, Japan). (b) Placement of a 5-Fr plastic stent.

ETGBD was selected to establish internal drainage through the demonstrated communication between the gallbladder and the subcapsular abscess cavity. This ERCP-based internal drainage procedure involves advancing a guidewire through the major duodenal papilla, common bile duct, and cystic duct into the gallbladder, followed by placement of a nasogallbladder tube or plastic stent. In the present case, the procedure was performed using an Olympus JF-260V duodenoscope (Tokyo, Japan). Initial attempts at cystic duct cannulation using a straight-tip EndoSelector guidewire (Boston Scientific, Marlborough, MA, USA) were unsuccessful, owing to the steep angle between the common bile duct and cystic duct preventing guidewire advancement. The device was subsequently exchanged for a novel Engetsu sphincterotome with adjustable tip direction via flexion and rotation (Kaneka Medix, Osaka, Japan), which facilitated successful guidewire insertion. A 5 Fr plastic stent was then deployed, achieving effective internal drainage (Figure 3).

**Figure 4:**
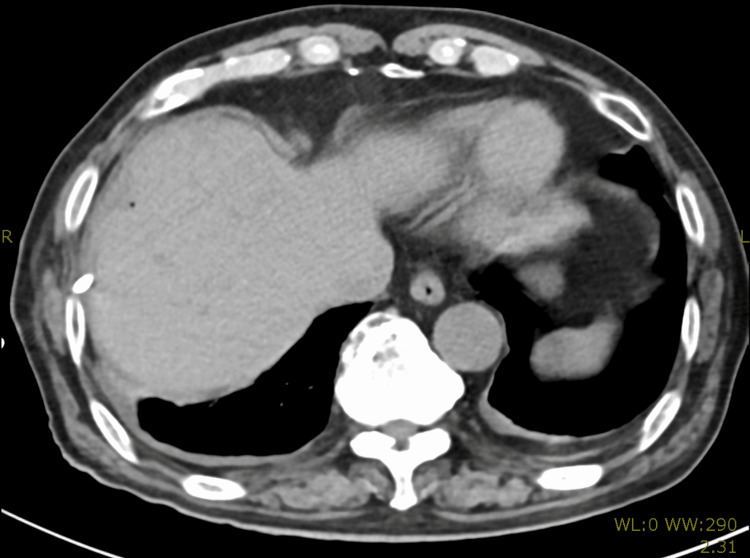
Computed tomography on hospital day 39 showing complete resolution of the subcapsular abscess.

The patient’s clinical course improved substantially following ETGBD, with CRP levels decreasing to 1.61 mg/dL by day 41 of his hospitalization (five days post-procedure). Follow-up cholangiography demonstrated resolution of the fistulous communication, and CT confirmed complete resolution of the subcapsular hepatic abscess (Figure 4).

The PTGBD catheter was subsequently clamped without cholecystitis recurrence before being ultimately removed. The patient remained clinically stable without recurrence, and was discharged on day 56.

Given the patient’s advanced age and persistent respiratory dysfunction, radical cholecystectomy was not planned. No recurrence or stent-related complications were observed over approximately three months of subsequent follow-up. The patient’s clinical course is summarized in Table 2.

**Table 2:** Timeline of the clinical course and drainage procedures MRCP: magnetic resonance cholangiopancreatography, PTGBD: percutaneous transhepatic gallbladder drainage, PTAD: percutaneous transhepatic abscess drainage, ETGBD: endoscopic transpapillary gallbladder drainage, CRP: C-reactive protein

Hospital day	Clinical event
Day 1	Admission with fever and right upper quadrant pain. CT and MRCP showed acute cholecystitis with gallbladder perforation, a subcapsular hepatic abscess, and communication between the gallbladder and abscess cavity. PTGBD was performed because surgery was contraindicated.
Days 2-21	Initial improvement with antibiotics and PTGBD. PTAD was not feasible because a safe access route could not be established due to intervening pulmonary and vascular structures.
Day 22	CRP decreased to 0.40 mg/dL.
Day 35	CRP increased to 5.89 mg/dL despite PTGBD, suggesting inadequate infection control.
Day 36	ETGBD was performed. A guidewire was advanced using an adjustable-tip sphincterotome, and a 5 Fr plastic stent was placed.
Day 39	Follow-up CT showed resolution of the subcapsular abscess.
Day 41	CRP decreased to 1.61 mg/dL.
Day 56	Discharged without recurrence.

## Discussion

Gallbladder perforation occurs in ~0.8-3.8% of patients with acute cholecystitis [8]. Its pathophysiology typically begins with persistent cystic duct obstruction, resulting in progressive intraluminal pressure and subsequent mucosal injury. Phospholipases released from the damaged mucosa promote intense inflammation and gallbladder wall necrosis with associated circulatory impairment [1]. Perforations most commonly occur at the gallbladder fundus, which has the poorest vascular supply [2]. In the present case, perforation occurred at the fundus adjacent to the hepatic bed, with inflammatory extension into the hepatic parenchyma that ultimately produced a subcapsular hepatic abscess. The subcapsular hepatic collection and its communication with the gallbladder were the key findings that suggested perforation with abscess formation.

Gallbladder perforation is classified, according to the Niemeier scale [9], into types I (chronic cholecystoenteric fistula), II (subacute localized perforation with pericholecystic abscess), and III (acute free perforation with generalized peritonitis). The present case was consistent with Niemeier type II.

As gallstones typically remain within the gallbladder in perforation-associated subcapsular hepatic abscesses, definitive surgical management is generally recommended to prevent recurrence and chronic inflammation. Consequently, successful non-operative management has rarely been reported [3]. In the present case, saline irrigation and cholangiography excluded catheter occlusion; however, spontaneous drainage remained insufficient. The most plausible explanation for this is that impaired gallbladder motility and firm adhesion between the gallbladder and liver limited effective decompression of the abscess cavity via PTGBD alone. ETGBD was therefore added to augment gallbladder decompression and establish internal drainage, given the clear anatomical communication between the gallbladder fundus and subcapsular abscess cavity. Because fistulous communication may evolve dynamically during the inflammatory process, this strategy should be considered only in carefully selected patients - particularly those in whom communication between the gallbladder and abscess cavity has been demonstrated or is strongly suspected, and in whom direct abscess drainage or surgery is anatomically difficult or considered clinically high-risk.

Miyagawa et al. previously described successful avoidance of surgery using combined PTGBD and endoscopic ultrasound (EUS)-guided abscess drainage in a patient with a gallbladder perforation and intra-abdominal abscess [3]. In that report, the abscess was located adjacent to the stomach, permitting a safe endoscopic puncture under EUS guidance. In our patient, conversely, the subcapsular hepatic abscess was situated beneath the anterior hepatic capsule, rendering both direct percutaneous and endoscopic drainage unsafe. We therefore leveraged the existing fistulous communication at the gallbladder’s fundus to achieve internal drainage, thereby concurrently addressing both the inflamed gallbladder and associated abscess cavity.

PTGBD is a well-established first-line option for acute cholecystitis, particularly in high-risk surgical patients, as it provides reliable gallbladder decompression through an external route. However, external drainage alone may be insufficient when adequate decompression of a communicating abscess cavity cannot be achieved. ETGBD offers internal drainage and may reduce dependence on prolonged external catheter management, although it is technically more demanding due to the need for selective cystic duct cannulation. EUS-guided gallbladder drainage represents another alternative in selected cases but requires a safe anatomical window and was not feasible in the present patient because of the subcapsular location of the abscess. Therefore, a combined PTGBD-ETGBD approach may be most suitable in selected patients with high operative risk, anatomically inaccessible abscesses for direct percutaneous drainage, demonstrated or strongly suspected communication between the gallbladder and abscess cavity, and inadequate infection control with PTGBD alone.

Although ETGBD is a well-established method for gallbladder drainage, with a prospective randomized study reporting a technical success rate of 86.1% and a per-protocol clinical success rate of 90.3% for EGBS [5], technical challenges related to cystic duct anatomy can hinder the procedure. Yoshida et al. classified technical ETGBD failures into five steps: inability to achieve papillary cannulation (Step 0); inability to identify the cystic duct orifice (Step 1); failure to advance the guidewire, owing to an unfavorable angle between the common bile duct and cystic duct (Step 2); inability of the guidewire to reach the gallbladder, owing to obstruction or tortuosity (Step 3); and failure to place a drainage catheter or stent, despite guidewire access (Step 4) [10]. In the present case, the steep angle between the common bile duct and the cystic duct corresponded to Step 2. According to a recent report, the novel Engetsu sphincterotome may facilitate selective guidewire insertion by allowing for finer adjustments of the tip’s direction [6]. Consistent with these observations, our use of the Engetsu device facilitated successful guidewire advancement and completion of ETGBD in the present case. Taken together, the temporal course and anatomical findings support a contributory role of ETGBD in this case, although the respective effects of ongoing antibiotic therapy and prior PTGBD cannot be fully separated in a single-case report. Further case accumulation is therefore warranted to clarify in which specific subgroups patients with gallbladder perforation and communicating abscess cavities this strategy may represent a feasible alternative to surgery.

## Conclusions

We achieved successful non-operative control of both gallbladder perforation and an associated subcapsular hepatic abscess in a patient with Niemeier type II perforation, in whom surgery was contraindicated due to severe respiratory dysfunction and direct percutaneous abscess drainage was anatomically infeasible. By combining PTGBD and ETGBD, we leveraged the fundal perforation as a conduit for internal drainage, thereby facilitating decompression of both the inflamed gallbladder and the associated abscess cavity and enabling sustained infection control. This combined approach may represent a useful therapeutic alternative in carefully selected high-risk surgical patients with gallbladder perforation and a communicating abscess cavity, particularly when both direct abscess drainage and surgery are not feasible.
